# IL-4 Up-Regulates MiR-21 and the MiRNAs Hosted in the CLCN5 Gene in Chronic Lymphocytic Leukemia

**DOI:** 10.1371/journal.pone.0124936

**Published:** 2015-04-24

**Authors:** Natalia Ruiz-Lafuente, María-José Alcaraz-García, Silvia Sebastián-Ruiz, Azahara-María García-Serna, Joaquín Gómez-Espuch, José-María Moraleda, Alfredo Minguela, Ana-María García-Alonso, Antonio Parrado

**Affiliations:** 1 Servicio de Inmunología, Hospital Clínico Universitario Virgen de la Arrixaca, Murcia, Spain; 2 Servicio de Hematología y Hemoterapia, Hospital Clínico Universitario Virgen de la Arrixaca, Murcia, Spain; 3 Universidad de Murcia, Murcia, Spain; 4 Centro de Investigación Biomédica en Red de enfermedades hepáticas y digestivas, Murcia, Spain; 5 Instituto Murciano de Investigación Biosanitaria Virgen de la Arrixaca (IMIB-Arrixaca), Murcia, Spain; IPMC, FRANCE

## Abstract

Interleukin 4 (IL-4) induces B-cell differentiation and survival of chronic lymphocytic leukemia (CLL) cells. MicroRNAs (miRNAs) regulate mRNA and protein expression, and several miRNAs, deregulated in CLL, might play roles as oncogenes or tumor suppressors. We have studied the miRNA profile of CLL, and its response to IL-4, by oligonucleotide microarrays, resulting in the detection of a set of 129 mature miRNAs consistently expressed in CLL, which included 41 differentially expressed compared to normal B cells (NBC), and 6 significantly underexpressed in ZAP-70 positive patients. IL-4 stimulation brought about up-regulation of the 5p and 3p mature variants of the miR-21 gene, which maps immediately downstream to the VMP1 gene, and of the mature forms generated from the miR-362 (3p and 5p), miR-500a (3p), miR-502 (3p), and miR-532 (3p and 5p) genes, which map within the third intron of the CLCN5 gene. Both genes are in turn regulated by IL-4, suggesting that these miRNAs were regulated by IL-4 as passengers from their carrier genes. Their levels of up-regulation by IL-4 significantly correlated with cytoprotection. MiR-21 has been reported to be leukemogenic, associated to bad prognosis in CLL, and the miRNA more frequently overexpressed in human cancer. Up-regulation by IL-4 of miR-21 and the miRNAs hosted in the CLCN5 locus may contribute to evasion of apoptosis of CLL cells. These findings indicate that the IL-4 pathway and the miRNAs induced by IL-4 are promising targets for the development of novel therapies in CLL.

## Introduction

The interleukin-4 (IL-4) pathway leads to maturation of B-cell precursors into immunoglobulin-secreting cells and antigen presenting cells, proliferation of activated B cells, and induction of isotype switching toward IgE [1]. IL-4 protects chronic lymphocytic leukemia (CLL) cells from spontaneous apoptosis or killing with DNA damaging agents [2–5]. CLL is a B-cell malignant disease most prevalent in the elderly, characterized by surface expression of the CD5 and CD23 markers, and a heterogeneous clinical course, with patients divided between those that never progress to late stages of the disease, and those that progress and require therapy. Prognostic markers such as IGVH status and ZAP-70 and CD38 expression levels are useful to evaluate the risk of progression [6]. Through its cytoprotective effect, the IL-4 pathway may sustain evasion of apoptosis of CLL cells, thereby contributing to leukemogenesis.

Binding of IL-4 to its surface receptor (IL-4R) induces phosphorylation of JAK1 and JAK3. JAK1 phosphorylates STAT6 which homodimerizes and enters the nucleus to regulate gene expression. JAK1 and JAK3 lead to anti-apoptotic signaling through PI3K/AKT and the mitochondrial pathway, and through the Ras/MAPK pathway and NFκB activation [7–11]. Recently, we have reported gene expression changes induced by IL-4 in CLL [12], but little is known about the response to IL-4 of microRNAs (miRNAs), an essential class of gene expression regulators.

Mature miRNAs are non-coding RNAs of 19–25 nucleotides in length, generated by processing of miRNA gene transcripts called pri-miRNAs. Based on their genomic localization, miRNAs can be divided into two main classes: intergenic, that constitute independent transcription units, and intragenic, located inside another gene and produced as part of the host gene mRNA [13]. The pri-miRNAs are capped and polyadenylated, then cropped by the Microprocessor complex, and the resulting stem-loop intermediate, called pre-miRNA, is exported to the cytoplasm. The pre-miRNA is further cleaved to generate miRNA duplexes in the RNA-induced silencing complex (RISC), where one or the other strand (5p or 3p) is degraded. The remaining strand, which constitutes the mature miRNA, is retained in the RISC and will target mRNAs by base-pairing to complete or partially complementary sites on the target mRNAs, usually located at the 3’ untranslated regions. As a consequence, gene expression is negatively regulated through mRNA degradation or, more commonly, translational repression. A single miRNA could repress expression of up to several hundred genes.

Deregulation of miRNAs has been implicated in human oncogenesis. In CLL, several miRNAs have been recurrently found overexpressed compared to normal B cells (NBC), such as miR-155 [14–19], miR-150 [14,16,19], miR-101 [14,18,19], miR-21 [14,18], miR-29a [18,19], or miR-29c [16,19], or underexpressed, such as miR-181a, miR-181b [15,18,19], and miR-223 [15,16,19]. CLL patients characterized by 13q14 or 17p deletions usually underexpress miR-15a [20,21] or miR-34a [16,22–25], located at the respective deleted regions, compared to other cytogenetic subtypes. Diverse animal models have illustrated the oncogenic potential of several miRNAs, including miR-155, miR-21, miR-29a, or the miR-17~92 cluster [26–29], and the tumor suppressor potential of others, such as the miR-15a/16-1 cluster [30,31]. MiRNA signatures frequently include higher expression of miR15a, miR-16, or miR-23b in patients expressing markers of worse prognosis, such as ZAP-70 [15,24,32,33]. In ZAP-70 negative patients miR-29a, miR-29b, miR-29c and miR-223 often show higher expression levels [15,16,19,32,33].

Here we report the identification of miRNAs regulated by IL-4 in CLL. MiR-21, miR-362, miR-500a, miR-502, and miR-532 were induced by IL-4, likely as a consequence of up-regulation of their respective host genes, vacuole membrane protein 1 (VMP1), and chloride channel, voltage sensitive 5 (CLCN5). MiR-21 is the most frequently overexpressed miRNA in human cancer, and could be involved in evasion of apoptosis and resistance to chemotherapy in CLL. IL-4 could exert its anti-apoptotic function through up-regulation of miR-21 and the microRNAs hosted in the CLCN5 gene. These findings may be useful in the development of therapeutic strategies targeting the IL-4 pathway in CLL.

## Methods

### Sample collection

Peripheral blood samples from 16 chronic lymphocytic leukemia (CLL) patients and 3 controls with normal lymphopoiesis were obtained. The study was approved by the Review Board of Hospital Clínico Universitario Virgen de la Arrixaca, and the participants provided their written informed consent. All the patients had leukocytosis and did not receive treatment in the last 3 months prior to sample collection (S1 Table).

### Cell isolation

Samples were processed to isolate the B cells by negative selection procedures which were based on cocktails containing CD2, CD16, CD36 and CD235a antibodies for depletion of T cells, NK cells, monocytes, macrophages, and erythrocytes. The RossetteSep Human B Cell Enrichment Cocktail kit (StemCell Technologies, Vancouver, Canada) was suitable for CLL samples, since these are rich in malignant B cells. This method is expensive but definitely worthwhile for cell populations present at relatively high levels. Small volumes of peripheral blood (10 mL) were collected from patients, and B cell isolation was directly performed during Ficoll 1.077 g/mL centrifugation, following the manufacturer’s instructions. However, due to the low content of B cells in the peripheral blood of normal subjects (usually less than 10% of the lymphocytes), larger volumes of peripheral blood were collected (500 mL). Thus, the use of the RossetteSep kit was not economically viable for normal samples. In addition, harvest of the fine, barely visible B cell layer formed at the interphase between diluted plasma and Ficoll is usually inefficient. An alternative method, the Dynabeads Untouched Human B cells kit (Invitrogen, Carlsbad, CA) was chosen for isolating NBC. Despite being more time consuming, this method is more efficient and less expensive. PBMC concentrates were obtained by centrifugation over Ficoll, and NBC isolated using the kit, following the manufacturer’s instructions. Enrichment was determined by labelling with CD19-FITC, CD3-PE, and CD5-PE-Cy7 (BD Biosciences), followed by acquisition in a FACScalibur flow cytometer (BD Biosciences), and analysis using the CellQuest software. Purity of CD19+ cells was 93.5±1.41% (mean±s.e.m.) in NBC and 97.43±0.62% in CLL, including 0.33±0.14% of CD19+CD5- potential normal B cells within the CLL fractions (range 0–1.05%). The percentage of ZAP-70 positive cells within the CD19+CD5+ fraction of CLL was determined from aliquots of peripheral blood subjected to red cell lysis, permeabilization with the Cytofix/Cytoperm kit, and labelling with CD19-FITC, ZAP-70-PE, CD5-PE-Cy7, and CD3-APC (BD Biosciences).

### Cell culture and determination of apoptosis

Following purification, three fractions of the purified CLL and NBC were processed: a) at time zero (“Pre”); b) after being cultured for 18 hours in RPMI-1640 medium supplemented with 10% fetal calf serum (Cambrex, East Rutherford, NJ) (“Ctrl”); and c) as b, but with adding 10 ng/mL of human recombinant IL-4 (BD Biosciences, San Diego, CA) (“IL-4”). Apoptosis of the cultured cells was determined by dual labelling with annexin V and propidium iodide (BD Biosciences), and flow cytometry analysis.

### RNA isolation

Total RNA, including RNA from approximately 18 nucleotides upwards, was isolated using the miRNeasy Mini Kit (Qiagen, Hilden, Germany). RNA samples were quantitated on a NanoDrop 2000 (Thermo Fisher Scientific, Whaltham, MA). RNA quality was examined on an Agilent 2100 Bioanalyzer (Agilent Technologies, Palo Alto, CA) using the RNA 6000 Nano Kit. Only samples with R.I.N. (RNA Integrity Number) >7.0 were further studied. Though the RNA 6000 Kit allowed to detect the presence of small RNA, this fraction was specifically analysed using the Small RNA Kit.

### Microarray analysis

From each RNA sample, 100 ng were labeled with cyanine 3-pCp (Cy3-pCp) using Agilent miRNA Complete Labeling and Hyb Kits, according to the manufacturer's protocol. The labeled miRNAs were hybridized onto Human miRNA Microarray Kit (V3, 8 × 15k) targeting 866 human and 89 human viral miRNAs. After hybridization, the microarray slides were washed and scanned in an Agilent G2565CA DNA Microarray Scanner. Images were analyzed with the Agilent Feature Extraction software. The automatically generated datasets were statistically analyzed and visualized using the GeneSpring GX software (Agilent). The miRNAs regulated by IL-4 were identified using the one-way ANOVA test (p<0.05) between samples Pre, Ctrl, and IL-4, with post hoc Tukey HSD analysis. The computation of p-values was performed using the Benjamini-Hochberg FDR correction. Those entities that passed the analysis with increases or decreases above 2-fold for comparisons IL-4 vs Pre, and IL-4 vs Ctrl concurrently and with the same direction of change were considered miRNAs regulated by IL-4. The same analyses were performed for comparisons between condition Pre vs all the others, and between condition Ctrl vs all the others. Our initial analysis performed with the first 8 patients showed that changes identified for comparison IL-4 vs all the others coincided with those identified for comparison IL-4 vs Ctrl using the Student’s t test. Though significant changes for comparison between condition Pre vs all the others were also identified (induced by cell culture), our main purpose was to identify miRNAs regulated by IL-4. For this reason, Pre sample was judged as dispensable from that time, and was not included in subsequent patients. Samples Ctrl and IL-4 from patient CLL03 did not pass the quality controls. Thus, our final analysis included 8 Pre, 15 Ctrl, and 15 IL-4 samples from 16 patients (S1 Table) and 3 paired Ctrl and IL-4 NBC samples. Datasets were deposited at the Gene Expression Omnibus database under accession number GSE62137. Hierarchical clustering analysis was performed using the Ward’s linkage method on euclidean distances. Fold changes of miRNAs regulated by IL-4 were compared between patients by Pearson correlation analysis using SigmaStat.

### Quantitative PCR (qPCR)

For miRNA expression, RNA samples were retrotranscribed with the miScript II RT Kit (Qiagen) using the miScript HiSpec buffer for mature miRNA detection only, followed by qPCR with the miScript SYBR Green PCR Kit (Qiagen) in an ABI Prism 7000 Sequence Detection System, using the miScript primer assays (Qiagen) for miR-21-3p, miR-362-3p, miR-362-5p, miR-500a-3p, miR-502-3p, miR-532, miR-532-3p, and RNU6-6P, the latter used as reference for normalization (cat. nos. MS00009086, MS00009562, MS00009569, MS00031920, MS00031927, MS00010052, MS00004571, and MS00033740, respectively). For mRNA expression, RNA samples were retrotranscribed with the iScript cDNA Synthesis Kit (Bio-Rad, Hercules, CA), followed by qPCR with the SYBR Premix Ex Taq (Takara Bio, Mountain View, CA), using QuantiTect primer assays (Qiagen) for VMP1, CLCN5, ZAP-70, and GAPDH, the two latter used as a validation of flow cytometry data [34], and as reference for normalization, respectively (cat. nos. QT00066241, QT00998683, QT00209251, and QT01192646, respectively). All these procedures were performed following the manufacturer’s instructions. Measures were performed in duplicate. Analysis with the 7000 System SDS software provided the cycle threshold (Ct) values. The Ct values for RNU6-6P or GAPDH were subtracted from the Ct values for the miRNAs and mRNAs, respectively, resulting in the ΔCt values. As RNA samples include the mRNA and miRNA fractions, GAPDH was also used for miRNA normalization. Next, the average ΔCt value for the Ctrl samples was subtracted from all the ΔCt values, resulting in the ΔΔCt values. Finally, the relative expression values, expressed as fold change compared to the average Ctrl samples, were generated using the formula 2^−ΔΔCt^. Individual fold changes for IL-4 samples compared to Pre and Ctrl samples were calculated as the ratios between their respective relative expression values.

### Semiquantitative RT-PCR

The levels of expression of VMP1 were measured using the GeneAmp RNA PCR Core Kit in a GeneAmp PCR System 9700 (both from Thermo Fisher Scientific), following the manufacturer’s instructions. First, RNA samples were retrotranscribed using random hexamers. Then, VMP1 sequences were amplified from the resulting RNA/cDNA hybrids using as primers the 5’-TTGTCCAGATGAAGAGGGCA-3’ and 5’-TCAAACATCCAGGACAACCAGT-3’ oligonucleotides, that map at exon 6 and exon 12, respectively, and 30 cycles of PCR. As reference, GAPDH sequences were amplified using the 5’-TCATGACCACAGTCCATGCC- 3’ and 5’- CATGAGGTCCACCACCCTGT-3’ oligonucleotides and 23 cycles of PCR. The amplified fragments were visualized following electrophoresis in agarose gels containing SYBR Safe stain (Thermo Fisher Scientific).

## Results

### MiRNA expression profile of CLL

**Fig 1:**
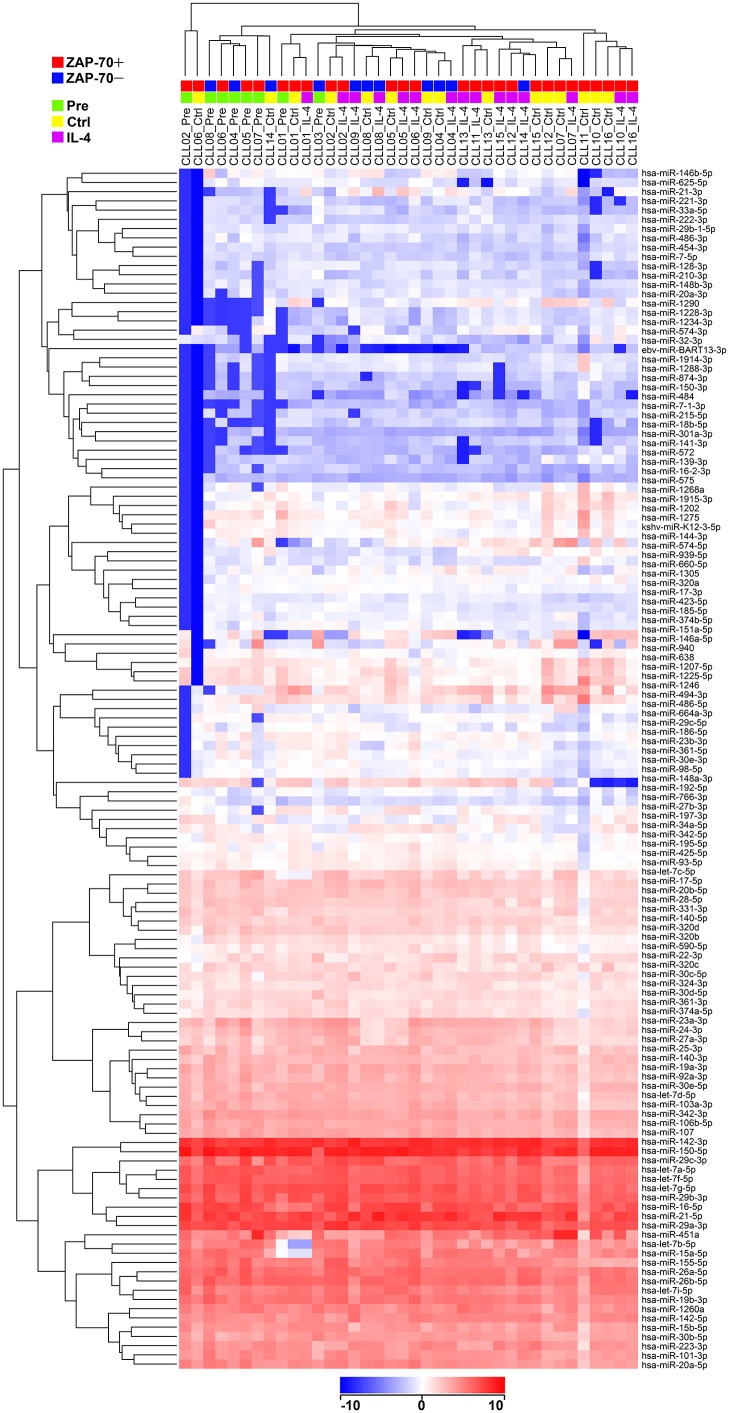
Hierarchical clustering analysis on the 129 miRNAs expressed in CLL using all the CLL samples. Microarray analysis was performed in 16 CLL patients, using different conditions: Pre (n = 8), Ctrl (n = 15), and IL-4 (n = 15), being all the Ctrl and IL-4 samples paired. The ZAP-70 status of the patients, positive (n = 11) and negative (n = 5), and the conditions of the samples, are indicated on the top. The relative levels of expression of miRNAs are depicted according to the shown log_2_ color scale on the bottom.

To define the set of miRNAs significantly expressed in CLL, we examined the cumulative distributions of mature miRNAs according to their signal intensity values in the arrays performed from the 8 Pre, 15 Ctrl, and 15 IL-4 CLL samples, which described an abrupt rise between the intensity values of −9 and −7, then a plateau up to −4, and a slow increase between −3 and 9 (S1 Fig). The cumulative distribution for the 3 Ctrl and 3 IL-4 NBC samples followed the same description with slight differences (S1 Fig). The absence of miRNAs significantly expressed between intensity levels of −7 and −4 led us to choose −4 as a positivity cut-off, which defined 129 and 149 miRNAs as significantly expressed in CLL (S2 Table) and NBC (S3 Table), respectively. Both lists share similarities, including the same top 10 miRNAs: miR-150-5p, miR-142-3p, miR-29a-3p, miR-21-5p, miR-16-5p, let-7g-5p, miR-29b-3p, let-7f-5p, miR-29c-3p, and let-7a-5p. However, 41 miRNAs were differentially expressed between CLL and NBC according to the Student t test (cut-off 2-fold, p<0.05), being 29 overexpressed in CLL, including miR-150-5p, miR-29a-3p, miR-29b-3p, let-7a-5p, miR-26a-5p, miR-451a, miR-155-5p, miR-101-3p, miR-28-5p, miR-144-5p, miR-486-5p, or miR-486-3p, and 12 underexpressed, including miR-181a-5p, miR-222-3p, miR-126-3p, miR-365a-3p, miR-181b-5p, miR-199a-3p, or miR-582-5p (Table 1). Hierarchical clustering analysis using conditions Pre, Ctrl, and IL-4, and the 129 miRNAs expressed in CLL, tended to segregate samples Pre from samples Ctrl and IL-4, and within the two latter, ZAP-70 positive from ZAP-70 negative (Fig 1). Moreover, comparison between ZAP-70 positive (n = 11) and negative (n = 5) patients by the Student t test (p<0.05) using the mean values of all the available samples from each patient, revealed a set of 6 miRNAs significantly underexpressed in ZAP-70 positive patients, with differences higher than 2-fold for miR-146b-5p, miR-210-3p, and miR-29c-5p, and higher than 1.5-fold for the ones with the highest levels of expression, miR-29c-3p and miR-30b-5p (Table 2). Comparisons between cytogenetic groups or untreated and previously treated patients were not performed because we did not have sufficient data in the cohorts. Clustering analysis also showed that expression was relatively stable across patients and conditions for most miRNAs. In addition, miRNAs expressed at similar levels grouped together, and the resulting miRNA sets were ordered according to their levels of expression (Fig 1). The 5p and 3p variants of a miRNA were often expressed at divergent levels and grouped in different sets, as is the case for miR-150-5p and miR-150-3p, or miR-21-5p and miR-21-3p. From the miR-17~92 cluster, miR-17-5p, miR-19a-3p, miR-19b-3p, miR-20a-5p, and miR-92a-3p were expressed at high levels, and miR-17-3p, and miR-20a-3p, at low levels. However, it was not a rule, since there were examples of paired 5p and 3p miRNAs expressed at similar levels, such as miR-142-3p and miR-142-5p, or miR-140-3p and miR-140-5p.

**Table 1:** MiRNAs diferentially expressed in CLL and NBC.

Mature miRNA ID	CLL*	NBC*	Fold change^†^	p value^§^
**Overexpressed in CLL**				
miR-150-5p	281.7	94.86	2.97	4.95E-03
miR-29a-3p	123.1	45.21	2.72	3.83E-04
miR-29b-3p	74.97	30.09	2.49	8.96E-04
let-7a-5p	62.11	29.72	2.09	4.64E-03
miR-26a-5p	44.06	17.78	2.48	7.87E-04
miR-451a	32.22	0.408	79.01	3.07E-06
miR-155-5p	26.28	6.397	4.11	1.11E-03
miR-101-3p	15.98	6.720	2.38	3.08E-03
miR-28-5p	3.593	0.400	8.99	4.98E-04
miR-140-5p	3.496	1.558	2.24	6.81E-05
let-7c-5p	3.280	1.519	2.16	4.84E-02
miR-374a-5p	2.422	0.538	4.51	8.46E-03
miR-320b	1.928	0.523	3.69	1.61E-02
miR-590-5p	1.759	0.320	5.50	2.66E-03
miR-34a-5p	1.267	0.458	2.77	2.04E-02
miR-195-5p	1.098	0.368	2.98	4.82E-02
miR-186-5p	1.088	0.315	3.45	2.88E-02
miR-144-3p	0.829	0.002	437.1	8.63E-06
miR-361-5p	0.757	0.233	3.26	2.50E-02
miR-23b-3p	0.754	0.198	3.81	3.56E-02
miR-30e-3p	0.713	0.205	3.47	2.87E-02
miR-192-5p	0.631	0.143	4.40	6.54E-03
miR-98-5p	0.622	0.182	3.41	2.13E-02
miR-486-5p	0.583	0.013	43.71	1.26E-04
miR-374b-5p	0.526	0.079	6.63	4.08E-03
miR-486-3p	0.276	0.013	21.7	2.30E-05
miR-128-3p	0.232	0.050	4.67	3.34E-02
miR-210-3p	0.202	0.022	9.32	1.22E-02
miR-141-3p	0.078	0.010	7.76	1.57E-02
**Underexpressed in CLL**				
miR-181a-5p	0.004	1.811	-417	3.10E-07
miR-222-3p	0.247	0.978	-3.96	2.15E-02
miR-126-3p	0.007	0.343	-50.7	2.80E-03
miR-365a-3p	0.017	0.318	-18.3	4.26E-02
miR-181b-5p	0.009	0.212	-24.9	1.94E-02
miR-199a-3p	0.007	0.198	-26.7	3.42E-03
miR-582-5p	0.002	0.195	-78.9	6.29E-09
miR-130a-3p	0.005	0.114	-21.6	1.79E-02
miR-155-3p	0.017	0.106	-6.12	4.86E-02
miR-501-5p	0.007	0.053	-8.11	9.73E-03
miR-132-3p	0.003	0.043	-13.4	1.25E-03
miR-30a-5p	0.006	0.041	-6.86	3.32E-02

*Relative mean values of 16 CLL and 3 NBC cases, whose values were calculated as the mean of all available samples (Pre, Ctrl, IL-4), except in the case of miRNAs modulated by IL-4, where these samples were excluded.

^†^CLL vs NBC.

^§^Student t test CLL vs NBC.

**Table 2:** MiRNAs diferentially expressed in ZAP-70 positive and negative patients.

Mature miRNA ID	ZAP-70 Pos*	ZAP-70 Neg*	Fold change^†^	p value^§^
hsa-miR-29c-3p	53.79	93.01	-1.73	4.7E-02
hsa-miR-30b-5p	11.98	17.93	-1.50	2.0E-02
hsa-miR-320b	1.740	2.418	-1.39	4.6E-02
hsa-miR-29c-5p	0.473	1.102	-2.33	8.3E-03
hsa-miR-146b-5p	0.184	1.464	-7.96	1.3E-02
hsa-miR-210-3p	0.144	0.432	-3.01	4.7E-02

* Relative mean values of 11 ZAP-70 Pos and 5 ZAP-70 Neg cases, whose values were calculated as the mean of all available samples (Pre, Ctrl, IL-4).

^†^ZAP-70 Pos vs ZAP-70 Neg.

^§^Student t test ZAP-70 Pos vs ZAP-70 Neg.

### Correlations between miRNAs and spontaneous apoptosis induced by cell culture

The levels of spontaneous apoptosis (Ctrl samples) and following IL-4 treatment were measured in 13 out of the 16 patients studied by microarray. The percentages of apoptotic cells were 28.9±4.01 (mean±s.e.m.) for Ctrl samples and 8.87±1.04 for IL-4 samples. The relationship between spontaneous apoptosis and baseline miRNA expression was examined by Pearson correlation analysis, resulting in significant negative correlations for 29 miRNAs, most of them expressed at high levels, including miR-29a-3p, let-7g-5p, miR-29b-3p, let-7f-5p, let-7a-5p, miR-26b-5p, miR-19b-3p, or miR-155-5p, and positive correlations for 9 miRNAs, all of them expressed at low levels, including miR-1246, or miR-638 (S4 Table). These results suggest that both groups may play anti- or pro-apoptotic roles, respectively. The ANOVA test with post hoc analysis for condition Pre vs conditions Ctrl and IL-4 (cut-off 2-fold, p<0.05) using all the available microarray experiments from the 16 patients, identified 12 mature miRNAs up-regulated by cell culture in CLL (S5 Table). From this list, miR-1246 and miR-1290 were positively correlated with spontaneous apoptosis, further suggesting that they are candidates to play pro-apoptotic roles in CLL.

### Identification of miRNA changes induced by IL-4 in CLL

**Fig 2:**
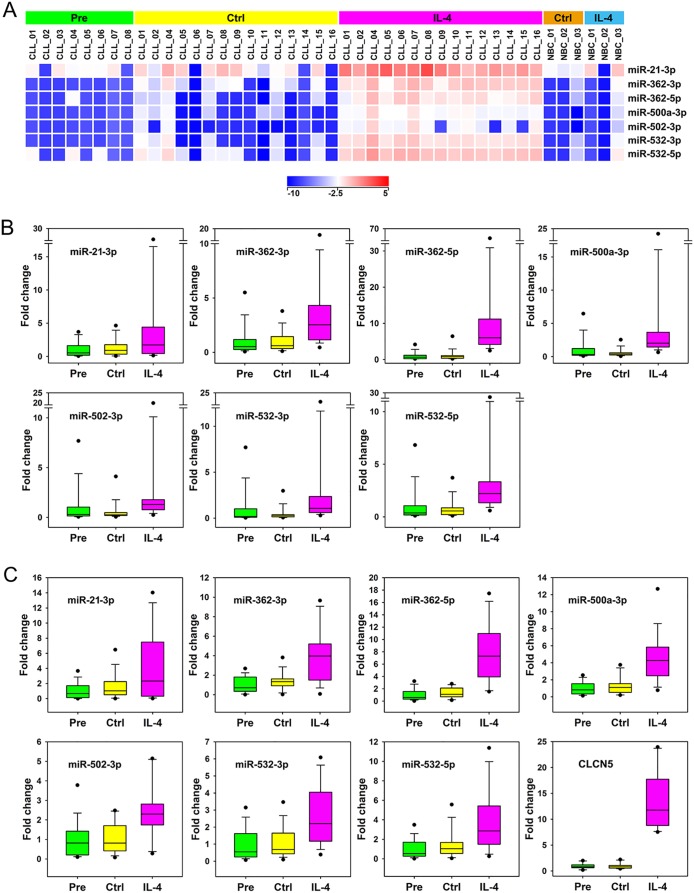
Identification of the miRNAs regulated by IL-4 in CLL. (A) Heat maps for expression of miRNAs significantly regulated by IL-4 in CLL (n = 15) following one-way ANOVA with Tukey HSD post hoc analysis for comparison of condition IL-4 vs Pre and Ctrl and above 2-fold change for both comparisons (p<0.05). Three NBC samples were included as controls. Relative expression levels are depicted according to the shown log_2_ color scale. (B, C) Validation of miRNAs regulated by IL-4 in CLL by qPCR, using RNU6-6P (B) and GAPDH (C) as references. Box whiskers representations of qPCR validations of the set of 7 miRNAs regulated by IL-4 in CLL are shown. Validation of up-regulation of the carrier gene CLCN5 by IL-4 in CLL, using GAPDH as reference, is included in C (right bottom panel). QPCR data are expressed as fold changes. For each assay, the average of Pre samples was set as 1. MiRNAs are ordered alphabetically.

The ANOVA test with post hoc analysis for condition IL-4 vs conditions Pre and Ctrl identified 7 mature miRNAs regulated by IL-4 in CLL (Fig 2A and S6 Table): miR-21-3p, miR-362-3p, miR-362-5p, miR-500a-3p, miR-502-3p, miR-532-3p, and miR-532-5p, all of them higher than 10-fold up-regulated on average. An identical set of 7 miRNAs had emerged in our first analysis, restricted to the 7 patients for which paired samples Pre, Ctrl and IL-4 were available (data not shown). Student paired t test for comparison IL-4 vs Ctrl (n = 15) identified an identical set of miRNAs and added to the list miR-21-5p (2.05-fold up-regulated), and miR-630 (9.95-fold down-regulated) (S6 Table). The latter was 94.6-fold higher for comparison Ctrl vs Pre, suggesting that IL-4, rather than inducing miR-630, partially counteracted its spontaneously induced up-regulation during cell culture. MiR-21 maps several hundred base pairs downstream of the last exon of VMP1, and miR-362, miR-500a, miR-502, and miR-532 within the third intron of CLCN5. Both are genes regulated by IL-4 according to our previous study [12], suggesting that regulation by IL-4 of these miRNAs is linked to that of their carrier genes. In general, their levels of change significantly correlated with each other (Table 3). In NBC, the whole set was up-regulated by IL-4 in 1 out of 3 cases (NBC_03) and miR-21-3p in another case (NBC_01) (Fig 2A).

**Table 3:** Correlations between the levels of cytoprotection and the amount of change in the levels of miRNAs by IL-4, and between the changes in the miRNAs with each other.

	Cytoprotection*	miR21-5p	miR21-3p	mi362-3p	mi362-5p	mi500a-3p	mi502-3p	mi532-3p	
R coefficient^§^	0.576								miR21-5p
p value	**3.95E-02**								
R coefficient	0.453	0.488							miR21-3p
p value	1.20E-01	9.07E-02							
R coefficient	0.311	0.655	0.679						mi362-3p
p value	3.02E-01	**1.51E-02**	**1.06E-02**						
R coefficient	0.559	0.621	0.528	0.625					mi362-5p
p value	**4.73E-02**	**2.35E-02**	6.36E-02	**2.24E-02**					
R coefficient	0.58	0.246	0.449	0.359	0.806				mi500a-3p
p value	**3.76E-02**	4.18E-01	1.23E-01	2.28E-01	**8.87E-04**				
R coefficient	0.444	0.412	-0.224	0.164	0.282	0.224			mi502-3p
p value	1.28E-01	1.62E-01	4.62E-01	5.93E-01	3.50E-01	4.62E-01			
R coefficient	0.548	0.585	0.542	0.639	0.996	0.834	0.283		mi532-3p
p value	5.26E-02	**3.58E-02**	5.55E-02	**1.86E-02**	**6.33E-13**	**3.95E-04**	3.48E-01		
R coefficient	0.493	0.636	0.654	0.918	0.829	0.619	0.223	0.841	mi532-5p
p value	8.68E-02	**1.95E-02**	**1.53E-02**	**9.41E-06**	**4.54E-04**	**2.41E-02**	4.65E-01	**3.16E-04**	

*Cytoprotection was calculated as the difference in apoptosis between conditions IL-4 vs Ctrl (n = 13)

^§^Pearson correlation analysis using fold changes between conditions IL-4 vs Ctrl expressed as log_2_ values; p values of less than 0.05 are typed in bold characters

### Validation of miRNAs and carrier genes regulated by IL-4 identified by microarray analysis

**Fig 3:**
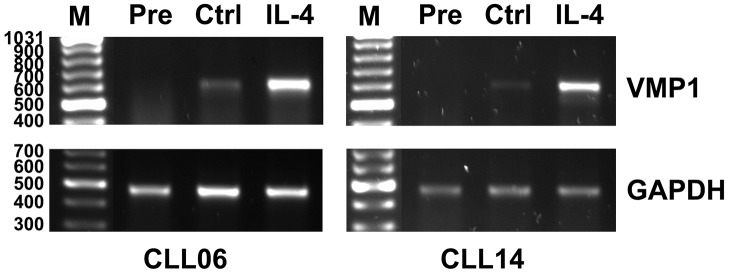
Validation of up-regulation of the carrier gene VMP1 by IL-4 in CLL. Two of the CLL patients, CLL06 and CLL14, were assayed by a semiquantitative RT-PCR assay. GAPDH was used as reference. The size of the molecular weight markers (M) is indicated to the left. The size of the expected PCR fragments were 606 bp for VMP1 and 464 bp for GAPDH.

The miRNAs regulated by IL-4 were assayed by qPCR, using RNU6-6P (Fig 2B) and GAPDH (Fig 2C) as references. ANOVA analysis for comparison of condition IL-4 vs conditions Pre and Ctrl confirmed the significant up-regulation of all the miRNAs included in this set (p<0.05, data not shown). The commercial qPCR assays used in this study confirmed up-regulation of CLCN5 by IL-4 in CLL (Fig 2C, right bottom panel), though not that of VMP1 (data not shown). Validation of VMP1 up-regulation by IL-4 in CLL was achieved by means of a semiquantitative RT-PCR assay (Fig 3).

### Correlations between ZAP-70 status, miRNA regulation by IL-4, and apoptosis

Correlation analyses between the levels of change of each miRNA and the baseline levels of expression of ZAP-70, and CD38, were performed to determine whether the miRNA response to IL-4 was related to CLL prognostic markers, but no significant correlations were found (data not shown). In contrast, the levels of cytoprotection correlated significantly with the levels of change of miR-21-5p, miR-362-5p, and miR-500a-3p (Table 3), providing additional evidence to suggest that these miRNAs could play a role in the anti-apoptotic response induced by IL-4 in CLL.

## Discussion

IL-4 is a key survival signal provided to CLL cells by the microenvironment. IL-4 induces profound gene and protein expression changes, which confer cytoprotection against cell death induced spontaneously or by cytotoxic drugs. Here, we have studied how IL-4 affects miRNA expression, an essential regulatory component of gene and protein expression regulation, which may help to understand the mechanisms sustaining CLL pathogenesis. By the use of a microarray platform which allows measuring the levels of expression of 955 mature miRNAs, we first identified 129 miRNAs expressed at significant levels in CLL. The miRNAs expressed at the highest levels coincided with those reported by previous studies, and were similar to those expressed by NBC in our study, though several divergences emerged between CLL and NBC, such as the previously reported overexpression of miR-150-5p, miR-29a-3p, miR-155-5p, or miR-101-3p, underexpression of miR-181a-5p, or miR-181b-5p [14–19], and others not firmly established yet, including the highly divergent miR-451a, miR-28-5p, miR-144-5p, miR-486-5p, or miR-486-3p, within the overexpressed, and miR-126-3p, miR-365a-3p, miR-199a-3p, or miR-582-5p, within the underexpressed.

Hierarchical clustering analysis grouped the Pre samples together, and Ctrl and IL-4 samples mixed, illustrating that cell culture on its own induces changes, as previously observed for gene expression [12]. Mixed Ctrl and IL-4 samples moderately grouped according to ZAP-70 status, indicating that this marker has a broad impact on miRNA expression in CLL. This is substantiated by differential expression of several miRNAs according to ZAP-70 status, including both the 3p and 5p variants of miR-29c, which is recurrently found down-regulated in ZAP-70 positive patients, miR-146b-5p, as in the recent study by Negrini et al [25], and miR-210-3p. These results support their potential use as prognostic biomarkers.

Correlation analysis between the baseline levels of miRNAs and spontaneous apoptosis indicated a relatively high number of candidate miRNAs with a potential anti-apoptotic or pro-apoptotic function. From this list, most miRNAs need functional validation, using a similar approach as that provided for the cytoprotective effects of miR-17-5p in the study by Bomben et al [35]. IL-4 is a strong anti-apoptotic stimulus for CLL cells, and the responsible mechanism has not been elucidated yet, though gene expression changes are likely involved. Many genes regulated by IL-4 have been proposed as potentially involved [12], though most of them will also require direct assessment. Because miRNAs control gene expression, it is possible that miRNAs regulated by IL-4 contribute to gene expression changes and to the anti-apoptotic function. We have found up-regulation of miR-21 (5p and 3p), miR-362 (3p and 5p), miR-500a-3p, miR-502-3p, and miR-532 (3p and 5p) by IL-4 in CLL. All these miRNAs were also up-regulated in at least 1 out of 3 NBC, indicating that their regulation by IL-4 is not restricted to CLL cells.

MiR-21 was previously found overexpressed in the allergic lungs of diverse asthma mice models (transgenic IL-4 and IL-13 mice, wild-type mice treated with allergen, IL-4 or IL-13) [36], and was induced by IL-4 in B cells [37]. MiR-21 is produced from two types of primary miR-21 transcripts, a pri-miR-21 arising from the miR-21 promoter located at the last introns of the VMP1 gene, and a VMP1-miR-21 transcript arising from the VMP1 promoter, both of which bypass the polyadenylation signals of VMP1 [38]. Because expression of VMP1 is induced by IL-4 [12], miR-21 up-regulation by IL-4 is likely produced through VMP1-miR-21 transcription. Concomitant up-regulation by IL-4 of the miRNAs comprised at the third exon of the CLCN5 gene suggests that they were likely produced also, rather than through transcription from their own promoters, as a consequence of being embedded within the CLCN5 primary transcript, another gene previously found up-regulated by IL-4 in CLL and NBC [12]. Thereafter, these primary transcripts would be successfully processed to produce the mature miRNAs.

MiR-21 is the miRNA most recurrently found overexpressed in human cancer [39], and its oncogenic role has been proven [27]. It has been found overexpressed in CLL [14,18], in association with promoter hypomethylation [40]. High level of expression of miR-21 associates to worse prognosis and chemotherapy resistance in CLL [41,42] and in cancer in general [43]. At the cellular level, an anti-apoptotic role has been proposed for miR-21, through repression of diverse pro-apoptotic targets, including PDCD4 and PTEN, resulting in activation of anti-apoptotic pathways such as Ras and NFκB [44]. Our data showed that miR-21-5p and some miRNAs hosted in the CLCN5 gene were significantly correlated to cytoprotection by IL-4 in CLL, further indicating these miRNAs as candidates to prevent cell death. This hypothesis should be directly assessed in future studies. Some of the miRNAs hosted in the CLCN5 gene have also been related to cancer, e.g., high expression of miR-362 is associated to worse prognosis and apoptosis resistance in colorectal and gastric cancer [45,46], that of miR-500a to hepatocellular carcinoma [47], and that of miR-502 to diverse types of cancer through a polymorphism in its target gene SET8 [48], and to colon cancer by inhibiting autophagy [49].

In summary, the present study identifies the miRNAs regulated by IL-4 in CLL and the probable responsible mechanism, contributing to the understanding of the anti-apoptotic response to IL-4, which could be relevant in evasion of apoptosis of CLL cells, resistance to chemotherapy, and leukemogenesis. Our data indicate that the IL-4 pathway and the miRNAs induced by IL-4 are promising targets for the development of novel therapies in CLL.

## Supporting Information

S1 Fig Cumulative distributions of mature miRNAs according to their signal intensity values. Microarrays performed on CLL and NBC were analysed with the flag “detected” set at “higher than 75%”. The absence of miRNAs significantly expressed between intensity levels of −7 and −4 led us to exclude values inferior to −7 as not distinguishable from background, and to choose −4 as a positivity cut-off, which defined 129 and 149 miRNAs as significantly expressed in CLL and NBC.
(TIF)

S1 Table Characteristics of CLL patients. (XLSX)

S2 Table MiRNAs expressed in CLL patients. (XLSX)

S3 Table MiRNAs expressed in NBC. (XLSX)

S4 Table Correlations between baseline expression of miRNAs and the levels of spontaneous apoptosis in CLL. (XLSX)

S5 Table MiRNA changes induced by cell culture not counteracted by IL-4 in CLL. (XLSX)

S6 Table MiRNA changes induced by IL-4 in CLL, and comparison with changes in NBC. (XLSX)
