# Supplementary figures and images for: IL-4 Up-Regulates MiR-21 and the MiRNAs Hosted in the CLCN5 Gene in Chronic Lymphocytic Leukemia
Source: PLoS One. 2015 Apr 24;10(4):e0124936. doi: 10.1371/journal.pone.0124936 (PMC4409305; doi:10.1371/journal.pone.0124936)

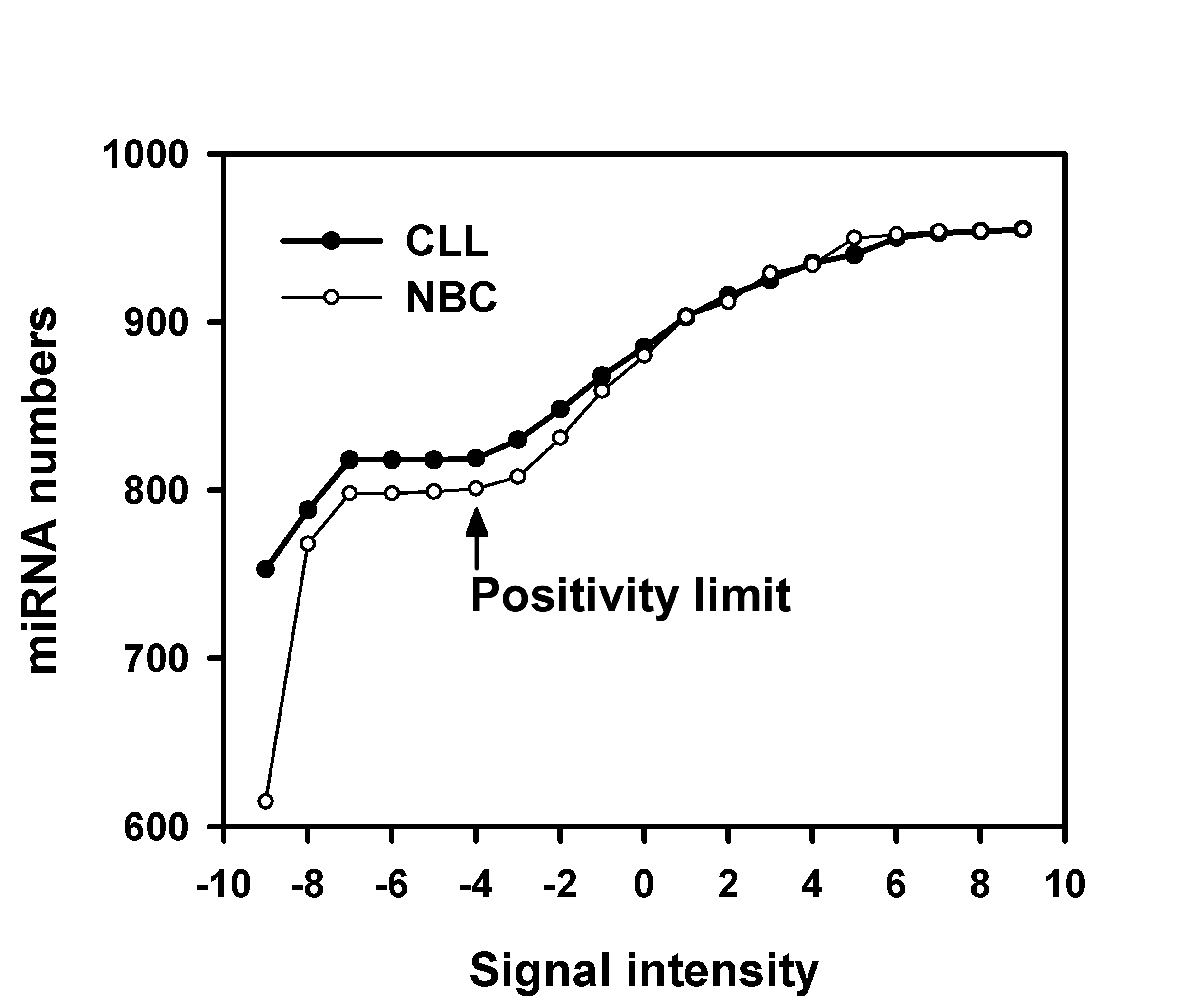

Supplement: S1 Fig — Microarrays performed on CLL and NBC were analysed with the flag “detected” set at “higher than 75%”. The absence of miRNAs significantly expressed between intensity levels of −7 and −4 led us to exclude values inferior to −7 as not distinguishable from background, and to choose −4 as a positivity cut-off, which defined 129 and 149 miRNAs as significantly expressed in CLL and NBC. (TIF) [file pone.0124936.s001.TIF]
